# Minimum variance beamforming combined with covariance matrix-based adaptive weighting for medical ultrasound imaging

**DOI:** 10.1186/s12938-022-01007-5

**Published:** 2022-06-18

**Authors:** Yuanguo Wang, Yadan Wang, Mingzhou Liu, Zhengfeng Lan, Chichao Zheng, Hu Peng

**Affiliations:** 1 School of Mechanical Engineering, Hefei University of Technology, 230009 Hefei, China; 2 Department of Biomedical Engineering, Hefei University of Technology, 230009 Hefei, China; 3 Anhui Province Key Laboratory of Measuring Theory and Precision Instrument, Hefei University of Technology, 230009 Hefei, China

**Keywords:** Minimum variance beamforming, Adaptive beamformer, Covariance matrix, Coherence, Standard deviation

## Abstract

**Background:**

The minimum variance (MV) beamformer can significantly improve the image resolution in ultrasound imaging, but it has limited performance in noise reduction. We recently proposed the covariance matrix-based statistical beamforming (CMSB) for medical ultrasound imaging to reduce sidelobes and incoherent clutter.

**Methods:**

In this paper, we aim to improve the imaging performance of the MV beamformer by introducing a new pixel-based adaptive weighting approach based on CMSB, which is named as covariance matrix-based adaptive weighting (CMSAW). The proposed CMSAW estimates the mean-to-standard-deviation ratio (MSR) of a modified covariance matrix reconstructed by adaptive spatial smoothing, rotary averaging, and diagonal reducing. Moreover, adaptive diagonal reducing based on the aperture coherence is introduced in CMSAW to enhance the performance in speckle preservation.

**Results:**

The proposed CMSAW-weighted MV (CMSAW-MV) was validated through simulation, phantom experiments, and in vivo studies. The phantom experimental results show that CMSAW-MV obtains resolution improvement of 21.3% and simultaneously achieves average improvements of 96.4% and 71.8% in average contrast and generalized contrast-to-noise ratio (gCNR) for anechoic cyst, respectively, compared with MV. in vivo studies indicate that CMSAW-MV improves the noise reduction performance of MV beamformer.

**Conclusion:**

Simulation, experimental, and in vivo results all show that CMSAW-MV can improve resolution and suppress sidelobes and incoherent clutter and noise. These results demonstrate the effectiveness of CMSAW in improving the imaging performance of MV beamformer. Moreover, the proposed CMSAW with a computational complexity of $O(N^2)$ has the potential to be implemented in real time using the graphics processing unit.

## Background

In medical ultrasound imaging, beamforming methods that form ultrasound image determine the diagnostic efficiency. The delay-and-sum (DAS) beamformer, which is simple and real-time, is widely implemented in most clinical ultrasound system to obtain ultrasound images. Nevertheless, it is susceptible to clutter and noise due to it is data-independent nature. The minimum variance (MV) beamformer was first studied as adaptive beamforming in ultrasound imaging [1, 2], showing a superior image resolution. Subsequently, various adaptive beamforming researches are focused on the MV beamformer [3, 4]. As MV can suppress interfering off-axis signals, improved visualization of heart tissue [5] and enhanced resolution in detecting microbubbles in super-resolution imaging [6] have been demonstrated. However, one of the main limitations of MV is the poor contrast which is similar to that of DAS beamformer.

Over the years, various studies have been attempted to overcome the limitation of MV in reducing incoherent noise. Eigenspace-based MV (ESBMV) [7] was developed to improve the contrast performance of MV by decomposing the covariance matrix into signal and noise subspaces. However, it tends to generate dark-region artifacts beside hyperechoic point-like targets. To deal with this issue, the eigenvalue threshold in ESBMV determined based on normalized spatial coherence [8] and normalized reciprocal of amplitude standard deviation [9] was successfully studied. Moreover, the forward–backward (FB) spatial smoothing technique was implemented in MV to increase the robustness [10]. Hasegawa et al. [11] proposed to build a cross-covariance matrix in MV using echo signals from different subarrays, which leads to a significant improvement in image contrast. Recently, Wang et al. [12] proposed a high-resolution MV based on optimal frequency-domain segmentation. In addition, a user parameter-free MV was developed [13] by adaptively determining the subarray length, number of samples for temporal averaging, and diagonal loading coefficient in MV.

Pixel-based adaptive weighting techniques have also been widely studied as an another solution to improve the imaging performance of MV. The coherence factor (CF) was studied to be combined with MV for medical ultrasound imaging, allowing improvements in both resolution and contrast with satisfactory robustness to sound velocity inhomogeneities [14]. The combination of CF and MV has also been demonstrated improved detection of residual bubbles [15]. Qi et al. [16] proposed to use the submatrix-based CF calculated using the MV beamformed output as a pixel-based adaptive weight for MV to improve the contrast without over-suppressing speckle signals. Additionally, the combination of CF and MV applied on the beamformed image data at different angles obtained a high-resolution image [17]. The generalized CF (GCF) [18] was also utilized to adaptively select the subarray length for spatial smoothing in MV to improve the speckle statistics without using the temporal smoothing technique [19]. The combination of MV and adaptive weighting have demonstrated effectiveness in improving the imaging performance of MV.

In addition to MV-based ultrasound beamforming, other adaptive beamforming methods include short-lag spatial coherence (SLSC) [20] and delay-multiply-and-sum (DMAS) [21] have been also widely studied to improve the image quality. SLSC has been studied in cardiac ultrasound imaging and demonstrated enhanced clutter reduction and endocardial border detection [22]. The transmit synthetic aperture extended the axial depth of field of SLSC imaging [23]. Nair et. al. proposed a robust SLSC (R-SLSC) which enables the use of higher lag information, and demonstrated that the R-SLSC provides increased contrast, signal-to-noise ratio, and contrast-to-noise ratio over traditional SLSC imaging [24]. The double-stage DMAS (DS-DMAS) [25] and regional-lag signed DMAS (rsDMAS) [26] were successfully developed to improve the imaging quality of DMAS. In addition, the DMAS has been studied to improve active contour segmentation in ultrafast ultrasound imaging [27] and improve contrast, resolution, and signal-to-clutter ratio by expanding the two-dimensional summations to four-dimensional summations [28]. Though effective in improving the resolution and contrast, the point detection of SLSC is high only when the channel signal-to-noise ratio (SNR) is low [29], and SLSC and DMAS both tend to generate dark-region artifacts beside hyperechoic point-like targets.

Recently, we introduced the covariance matrix-based statistical beamforming (CMSB) [30] for medical ultrasound imaging to reduce sidelobes and incoherent clutter and noise. The CMSB estimates adaptive weights for coherent summation on a modified covariance matrix estimated using adaptive spatial smoothing, rotary averaging, and diagonal reducing using the mean-to-standard-deviation ratio (MSR). Improved resolution and contrast over conventional DAS have been demonstrated. Therefore, we hypothesize that an appropriate integration of CMSB and MV has the potential to improve the contrast and resolution of MV.

In this paper, we proposed a new adaptive weighting approach for MV beamformer based on CMSB to enhance the imaging performance. The proposed method estimates the MSR of a modified covariance matrix reconstructed by adaptive spatial smoothing, rotary averaging, and diagonal reducing. Furthermore, we adaptively determine the diagonal reducing load based on the aperture coherence with the aim to further preserve speckle and enhance detection of anechoic and hypoechoic cysts. Simulation, experimental, in vivo rat mammary tumor, and human heart studies were conducted to validate the performance of the proposed method.

The rest of this paper is organized as follows. The results are given in “Results” section. We discuss on the proposed method in “Discussion” section, and draw conclusions in “Conclusion” section. In “Methods” section, we briefly introduce the data model of synthetic aperture imaging, DAS, MV, and the proposed method in detail, and present the simulation, experimental, and in vivo datasets as well as evaluating metrics.

## Results

### Simulation results

**Fig. 1:**
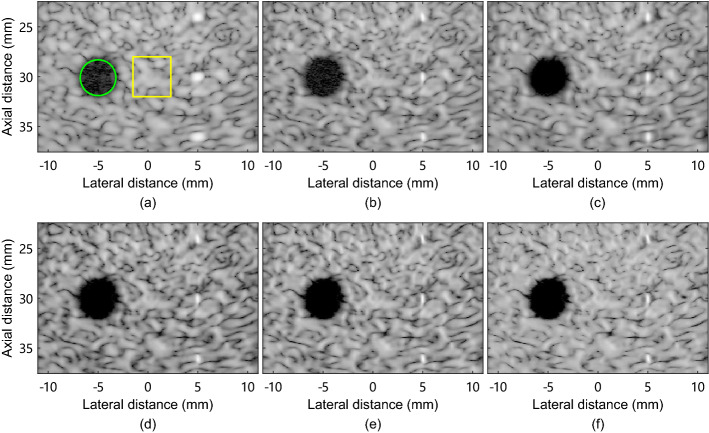
Simulated tissue-mimicking phantom images reconstructed by **a** DAS, **b** MV, **c** ESBMV, **d** GCF-MV, **e** CMSF-MV, and **f** CMSAW-MV. All images are shown in a 60-dB dynamic range

Figure 1 shows simulated tissue-mimicking phantom images reconstructed using different methods. The noise and artifacts inside the cyst target can be clearly seen in DAS and MV images. It can be seen that ESBMV image shows improved contrast with reduced noise inside the cyst, compared with MV. GCF-MV image shows improved contrast compared with MV image, but black-spot artifacts are introduced, leading to decreased speckle intensity. CMSF-MV and CMSAW-MV images show improved contrast over MV image. Besides, the background speckle pattern in CMSAW-MV image is smoother than that in CMSF-MV image, indicating a higher speckle retaining performance.

**Fig. 2:**
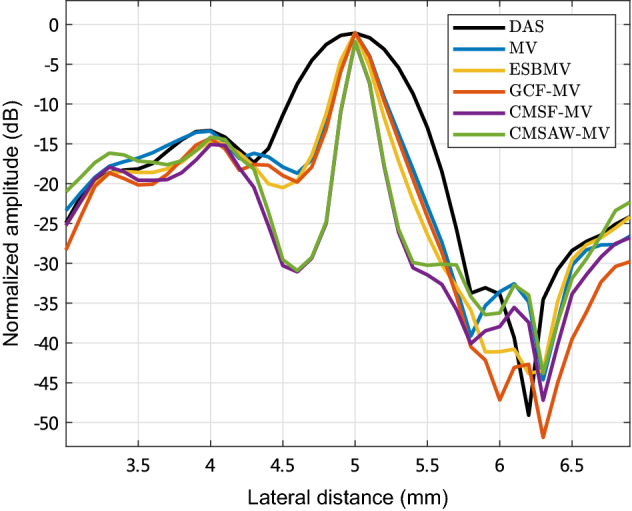
Lateral variations through the point target at 24 mm depth in simulated images reconstructed by different methods

Figure 2 plots the lateral variations through the point target at 24 mm depth in simulated images. We can see that MV provides a narrower mainlobe width compared with DAS. ESBMV and GCF-MV have nearly the same mainlobe width in comparison with MV, indicating no resolution improvement. Moreover, CMSF-MV and CMSAW-MV both obtain the narrowest mainlobe width, which indicates the improved resolution over MV.

Table 1 lists FWHM, CR, gCNR, and sSNR of simulated images formed using different methods. Compared with MV, ESBMV improves the contrast by 37.5%. Although GCF-MV provides contrast improvement of 51.6% over MV, it retains the resolution and decreases the gCNR and sSNR by 13.1% and 13.0%, respectively. CMSF-MV and CMSAW-MV improve the average resolution by 38.7% compared with MV. CMSAW-MV achieves improvements of 59.4%, 11.4%, and 14.5% in CR, gCNR, and sSNR. This indicates that the proposed CMSAW-MV enhances the imaging performance in terms of resolution, contrast of anechoic cyst, and speckle statistics over MV.

### Experimental results

**Fig. 3:**
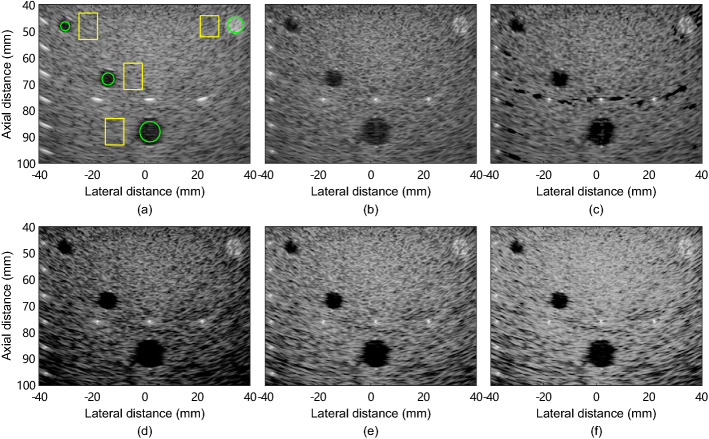
Experimental tissue-mimicking phantom images reconstructed from the dataset *geabr_0* by **a** DAS, **b** MV, **c** ESBMV, **d** GCF-MV, **e** CMSF-MV, and **f** CMSAW-MV. All images are shown in a 60-dB dynamic range

Figure 3 shows experimental phantom images reconstructed from the dataset *geabr_0* using different methods. We can see that MV image shows improved resolution and degraded contrast compared with DAS image. Although ESBMV image shows improved contrast over MV image, dark-region artifacts appears visually beside hyperechoic targets. GCF-MV image shows improved contrast compared with MV image, but the speckle intensity degrades and the variations in speckle regions at all imaging depths are larger. As seen from Fig. 3(e)-(f), CMSF-MV and CMSAW-MV improve the contrast over MV while simultaneously enhance the intensity and smoothness of the speckle pattern. Compared with CMSF-MV image, CMSAW-MV image shows smoother speckle pattern and improved speckle intensity. It should be noted that CMSF-MV and CMSAW-MV images show degraded visualization of the hyperechoic cyst in comparison with MV image, indicating a degraded contrast performance for hyperechoic cyst.

**Fig. 4:**
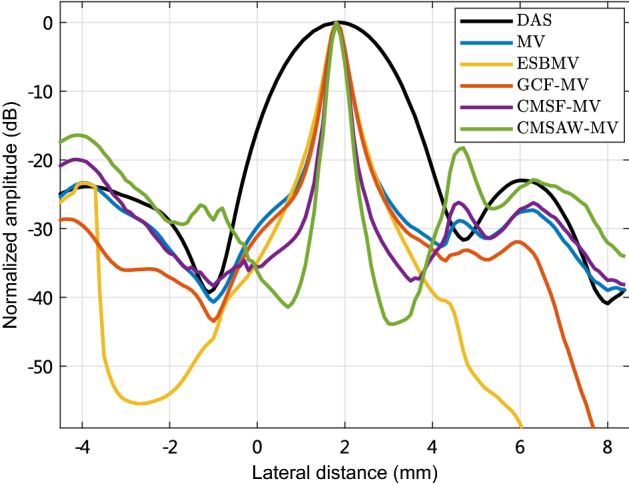
Lateral variations through the point target located at ($x=1.8$ mm, $z=75.9$ mm) in phantom experimental images reconstructed by different methods

Figure 4 plots the lateral variations through the point target located at ($x=1.8$ mm, $z=75.9$ mm) in the experimental images. We can see that MV obtains a narrower mainlobe compared with DAS, and ESBMV and GCF-MV obtain nearly the same mainlobe width in comparison with MV. In addition, CMSF-MV and CMSAW-MV obtain the narrowest mainlobe width, and this indicates the improved resolution.

Lateral FWHM, CR, gCNR, and sSNR of experimental images formed using different methods are listed in Table 2. The average FWHM was estimated using the three point targets at various depths. It can be found that DAS obtains the worst resolution, but its sSNR is higher than that of MV, ESBMV, GCF-MV, and CMSF-MV. In contrast, MV significantly improves the resolution, but provides lower contrast, gCNR, and sSNR. ESBMV mainly improves the contrast of MV. Moreover, GCF-MV obtains the same resolution and improves the average CR by 105.7% and 9.6% for anechoic cysts and hyperechoic cyst, respectively, compared with MV, but its gCNR for anechoic cysts and sSNR degrade. In comparison with MV, CMSF-MV obtains improvements of 21.3%, 107.6%, and 89.2% in resolution, average CR and gCNR for anechoic cysts, respectively, whereas the sSNR is 17.9% lower than that of MV. In addition, CMSAW-MV achieves improvements of 21.3%, 115.9%, and 138.2% in resolution, average CR and gCNR for anechoic cysts, but the average sSNR degrades by 2.2%. Note that the proposed CMSF-MV and CMSAW-MV degrade the contrast for hyperechoic cyst compared with MV.

**Fig. 5:**
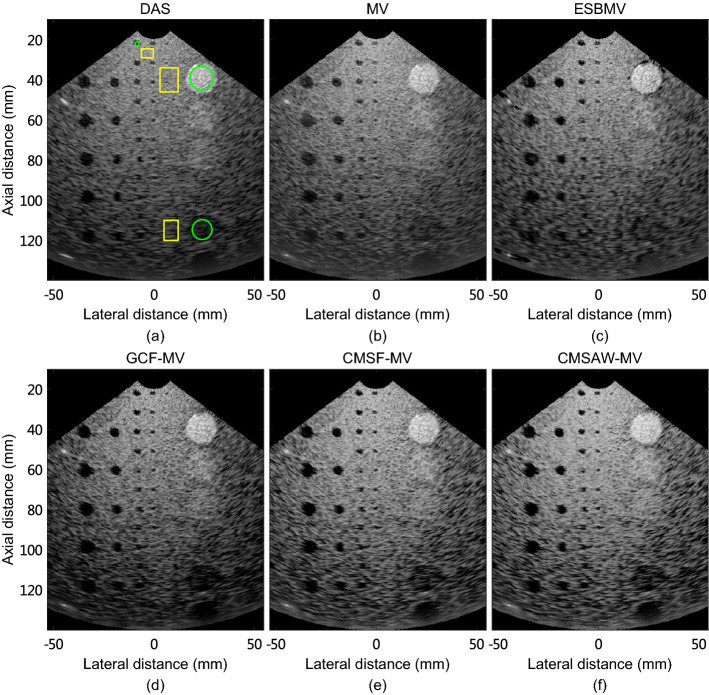
Phantom experimental images reconstructed from the dataset *ats* by **a** DAS, **b** MV, **c** ESBMV, **d** GCF-MV, **e** CMSF-MV, and **f** CMSAW-MV. All images are shown in a 60-dB dynamic range

Figure 5 shows the experimental phantom images reconstructed from the dataset *ats* using different methods. We can see that ESBMV and GCF-MV both improve the contrast of anechoic cysts. The dark-region artifacts appear beside point targets in ESBMV image, and the speckle intensity at the large depth degrades significantly in GCF-MV image. In addition, CMSF-MV and CMSAW-MV obtain improved contrast for anechoic and hypoechoic targets in comparison with MV, and at the same time retain the speckle intensity at the large depth.

Table 3 lists the CR, gCNR, and sSNR of anechoic, hypoechoic, and hyperechoic cysts at depths of 22, 114.5, and 39.2 mm in phantom experimental images formed from dataset *ats* using different methods. Compared with MV, GCF-MV improves contrast improvements of 62.7%, 100.0%, and 9.6% for anechoic, hypoechoic, and hyperechoic cysts, respectively. In comparison with MV, CMSF-MV obtains contrast improvements of 69.1% and 89.1% for anechoic and hypoechoic cysts, respectively, but degrades the contrast by 12.8% for hyperechoic cyst. CMSAW-MV improves the contrast by 76.8% and 113.0% for anechoic and hypoechoic cysts, respectively, over MV, and 8.7% and 6.5% for anechoic and hypoechoic cysts, respectively, over GCF-MV. However, the contrast for hyperechoic cyst obtained by CMSAW-MV degrades by 12.7% compared with MV. In addition, CMSAW-MV obtains the highest gCNR for anechoic and hypoechoic targets among all methods.

### In vivo rat mammary tumor

**Fig. 6:**
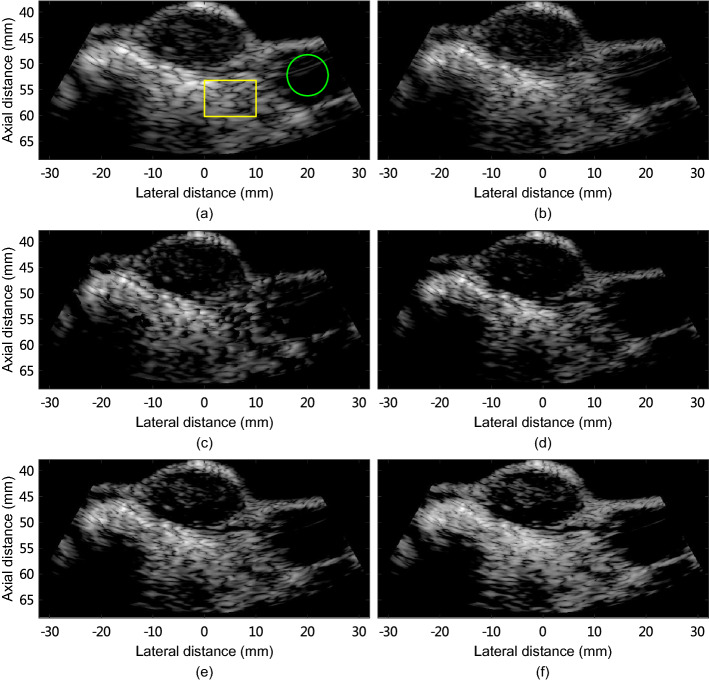
Images of the rat mammary tumor reconstructed by **a** DAS, **b** MV, **c** ESBMV, **d** GCF-MV, **e** CMSF-MV, and **f** CMSAW-MV. All images are shown in a 50-dB dynamic range

Figure 6 shows the rat mammary tumor images reconstructed using different methods, and Table 4 lists CR, gCNR, and sSNR obtained by different methods. These metrics were calculated using the regions indicated by the green and yellow boxes depicted in Fig. 6(a).

ESBMV provides improved contrast compared with MV, but damages the tissue texture. Besides, GCF-MV reduces clutter and noise compared with MV but suppresses the background tissue. CMSF-MV and CMSAW-MV both improve the contrast and preserve the tissue compared with GCF-MV. In addition, CMSAW-MV provides a better smoothed tissue compared with CMSF-MV, and achieves the best detection of hypoechoic masses inside the tumor. ESBMV obtains lower gCNR and sSNR compared with MV. GCF-MV improves the CR by 74.0% over MV, but the gCNR and sSNR are decreased by 31.9% and 5.4%, respectively. Compared to MV, CMSF-MV improves the contrast, gCNR, and sSNR by 69.6%, 7.2%, and 20.3%, respectively. CMSAW-MV provides improvements of 6.0%, 14.9%, and 31.5% in CR, gCNR, and sSNR over CMSF-MV, respectively, showing enhanced performance in improving contrast and preserving tissue texture.

### In vivo human heart

**Fig. 7:**
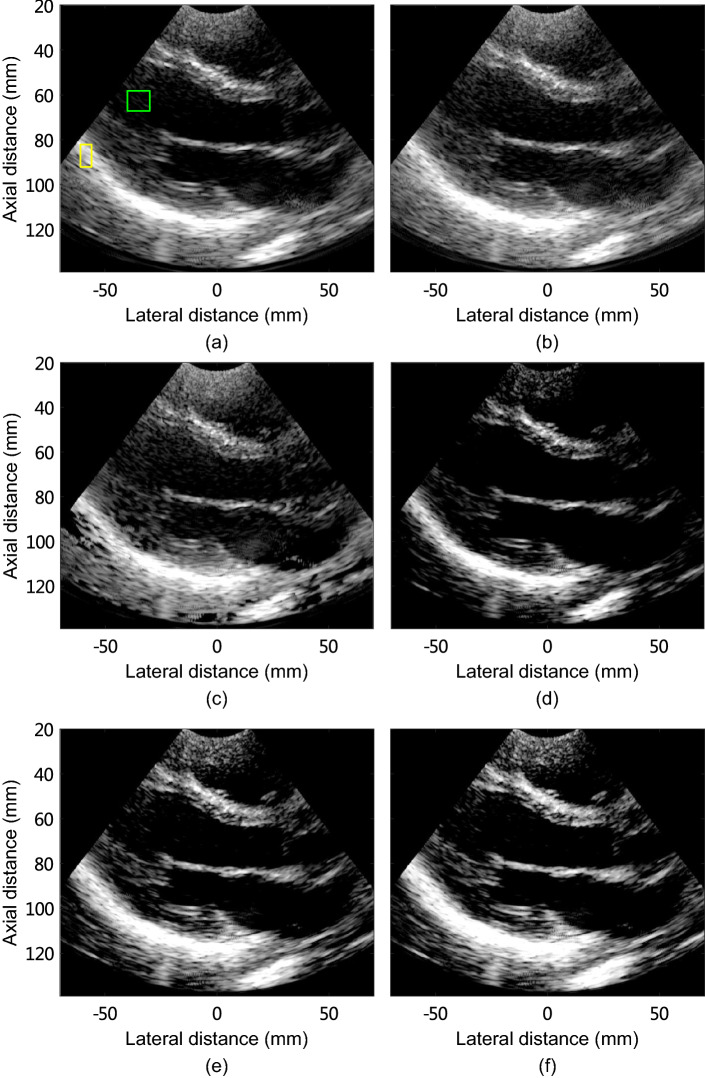
Images of the human heart reconstructed by **a** DAS, **b** MV, **c** ESBMV, **d** GCF-MV, **e** CMSF-MV, and **f** CMSAW-MV. All images are shown in a dynamic range from -60 dB to -15 dB

The in vivo human heart images reconstructed using different methods are shown in Fig. 7. The CR, gCNR, and sSNR were calculated to evaluate noise suppression and tissue preservation performances of the proposed method and are listed in Table 5. These metrics were calculated using the regions indicated by the green and yellow boxes depicted in Fig. 7a. As seen in Fig. 7b, MV image shows higher noise level compared with DAS image. ESBMV image also shows the background noise, and the tissue is removed to some extent. Compared with MV image, GCF-MV image shows obviously reduced background noise. Additionally, CMSF-MV and CMSAW-MV images show significantly reduced noise compared with MV, and enhanced tissue preservation over GCF-MV image.

As illustrated in Table 5, MV provides a degradation of 3.2 dB in contrast compared with DAS. ESBMV provides degraded contrast, gCNR, and sSNR compared with MV. It should be noted that this is not consistent with the results in simulation, phantom experimental, and rat mammary tumor studies. Compared with MV, GCF-MV improves the contrast by 33.0% but obtains degradation of 32.1% and 30.1% in gCNR and sSNR, respectively. In addition, CMSF-MV improves the contrast and gCNR by 27.9% and 4.8%, respectively, over MV, whereas the obtained sSNR is 2.7% lower than that obtained by MV. Compared to CMSF-MV, CMSAW-MV obtains improvements of 7.2%, 9.1%, and 20.2% in contrast, gCNR, and sSNR, respectively. This indicates that CMSAW-MV significantly improves the contrast, gCNR, and sSNR compared to MV. The human heart results show that the proposed methods are well suitable for tissue imaging.

## Discussion

The novelty of this work is that we introduce an adaptive weighting approach based on CMSB to address the contrast issues of the MV beamformer, and improve resolution as well. From this, aperture coherence based adaptive diagonal reducing is introduced with the aim to improve the speckle preservation. We applied the proposed method on the simulated, experimental, in vivo rat mammary tumor, and human heart datasets. The results all confirm the effectiveness of the proposed CMSF-MV and CMSAW-MV in enhancing ultrasound image quality. We discuss the results in the following in detail.

The simulation and phantom experimental results indicate that the proposed CMSF-MV and CMSAW-MV both obviously enhance the resolution and contrast performances of MV, leading to a higher contrast of MV over that of ESBMV as illustrated in Tables 1, 2. It is noted that CMSF-MV obtains almost retained gCNR and slightly lower sSNR compared with MV in simulation, whereas it obtains improved gCNR and degraded sSNR in phantom experiments. In contrast to MV, the proposed CMSAW-MV achieves improvements in resolution, contrast, gCNR, and sSNR in both simulation and experiment, showing comprehensive improvement in imaging performance.

The in vivo rat mammary tumor and human heart studies also demonstrate the effectiveness of the proposed methods on improving the imaging performance of MV. According to Table 4, CMSF-MV and CMSAW-MV both improve the contrast, gCNR, and sSNR over MV, showing enhanced lesion detectability and detection of hypoechoic mass in the tumor region at the depth of 45 mm. Besides, the proposed CMSF-MV and CMSAW-MV enhance the visualization of heart tissue as illustrated in Table 5. This indicates that the proposed methods are suitable for tissue imaging focused at detecting lesion (e.g., breast imaging).

It is worth noticing that MV introduces background noise in the human heart study as seen in Fig. 7b, and ESBMV cannot reduce the noise effectively (Fig. 7c). This illustrates that MV has a poor ability to reduce noise in in vivo heart imaging.

Compared to CMSF-MV, CMSAW-MV provides higher imaging performance in terms of image contrast and speckle smoothness. This is owing to the adaptive selection of the diagonal reducing factor $\delta$ based on the aperture coherence. It is worth mentioning that the resolution, contrast, lesion detection, and speckle statistics of the MV beamforming are all significantly enhanced through the CMSAW weighting.

Although CMSAW-MV obtains a significant improvement in sSNR over MV in simulation, in vivo rat mammary tumor, and human heart studies, it provides a lower sSNR at the depth of 88 mm in phantom experiment. This is likely because the amplitude standard deviation used for the selection of subarray length is susceptible to the echo signals with low SNR from a large imaging depth, which causes degradation in retaining speckle signals. However, the combination of the proposed CMSAW and the modified MV with adaptive spatial smoothing [19], which significantly improves the speckle statistics, has the potential to overcome this problem. Another limitation of CMSF-MV and CMSAW-MV is the degradation of imaging on hyperechoic targets. As seen from Fig. 3, the contrast for hyperechoic cyst in CMSF-MV and CMSAW-MV degrades, which is 19.5% and 32.0% lower than that of MV. This is likely because the dynamic subarray lengths for hyperechoic region tend to be small due to the off-axis scattering. Hence, a part of small weight values are generated for hyperechoic signals, which leads to degraded contrast for hyperechoic targets.

As the proposed CMSAW for MV takes into account the speckle preservation performance, it is suitable for tissue imaging. Furthermore, the CMSAW can be extended using modified MV methods to further improve the image quality, and this will be studied in the future. It should be noted that the proposed CMSAW has the potential to be combined with conventional DAS beamforming and other advanced ultrasound beamforming methods, for example, DMAS and multi-covariate imaging of sub-resolution targets (MIST) [31], to improve the resolution.

Consider a linear transducer with *N* elements, the computational complexity of the standard MV beamforming is quite high because of the inversion of the covariance matrix, which is $O(L^3)$. There are several ways to address this issue. On one hand, low complexity MV beamformers [32, 33] can be implemented to reduce the computational complexity. On the other hand, the implementation of MV on graphics processing unit (GPU) [34, 35] can be utilized to handle the large computational load and achieved real-time calculation. Furthermore, the neural network MobileNetV2 can be used to speed up MV beamforming by reducing the number of parameters and computational complexity [36]. The spatial smoothing for estimation of covariance matrix in CMSF and CMSAW requires a computational complexity of $O({L^{'}}^2)$. The dynamic selections of dynamic subarray length $L^{'}$ and diagonal reducing factor $\delta _{ac}$ require a computational complexity of *O*(*N*), and rotary averaging, diagonal reducing, and estimation of MSR on the modified covariance matrix require a computational complexity of $O({L^{'}}^{2})$. Since $L^{'}$ is typically less than or equal to *N*/2, the computational complexity of CMSF and CMSAW is about $O(N^{2})$. Therefore, the proposed CMSF-MV and CMSAW have the potential to be implemented in real time since MV with higher computational complexity has been successfully implemented in real time with GPU .

## Conclusion

We have proposed a novel adaptive weighting approach for MV beamformer based on CMSB to improve the imaging performance in STA ultrasound imaging. The proposed method was implemented and validated using simulated, experimental, and in vivo data. These results demonstrate the effectiveness of the proposed method in improving resolution, contrast, and lesion detection of the MV beamformer. To conclude, the proposed approaches can provide significantly improved image quality with acceptable increased computational load. Besides, the proposed approaches have the potential to be implemented in real time.

## Methods

### Data model of synthetic aperture imaging

The synthetic transmit aperture (STA) ultrasound imaging as an ultrasound imaging modality, can achieve two-way focusing, and thus obtains a high resolution [37]. We used the STA imaging as the imaging mode for evaluating the imaging performance of ultrasound beamformers in this study. In STA imaging, channel signals are acquired by insonifying a phantom using an un-focused spherical wave using only one element. Consider a linear transducer array with *N* elements used to transmit and receive. After the channel signals at imaging point *p* are delay-compensated, a two-dimensional full SA data matrix is given by

1 $$\begin{aligned} \small \mathbf {\varvec{X}}(p) = \left[ \begin{array}{cccc} x_{1,1}(p) &{} x_{1,2} (p) &{} \cdots &{} x_{1,N}(p) \\ x_{2,1}(p) &{} x_{2,2}(p) &{} \cdots &{} x_{2,N}(p) \\ \vdots &{} \vdots &{} \ddots &{} \vdots \\ x_{N,1} (p) &{} x_{N,2}(p) &{} \cdots &{} x_{N,N}(p). \\ \end{array} \right] \end{aligned}$$

Through focusing in receive and transmit apertures using conventional DAS beamforming, we obtain the final high-resolution output as

2 $$\begin{aligned} \begin{aligned} Y _\mathrm {DAS}(p)= \sum _{i=1}^{N}{w_{i}}\sum _{j=1}^{N}{w_{j}x_{ij}(p)}= \sum _{i=1}^{N}{w_{i}{\mathbf {x}}_{i}(p)}, \end{aligned} \end{aligned}$$

where *p* is the index of imaging point, and $x_{ij}(p)$ represents the signal sample received by the *j*th element in the receiving array on *i*th emission at the imaging point *p* after delay compensation. ${\mathbf {x}}(p)=[{\mathbf {x}}_1(p), {\mathbf {x}}_2(p), ... , {\mathbf {x}}_N(p)]^T$ is the receive aperture synthesized output. $w_{j}$ and $w_{i}$ represent the predefined weight for *j*th reception and *i*th transmission, respectively.

In this study, the delay-compensated channel signals are first beamformed in the receive aperture using the DAS beamforming, and the receive-synthesized data are obtained. Then, adaptive beamforming methods are applied on the receive-synthesized data to generate the beamformed output.

It should be noted that the *f*-number and apodization window are important for DAS beamforming to generate an optimum image quality in resolution and contrast [38]. Therefore, the *f*-number and apodization should be appropriately chosen to obtain an optimum DAS image.

### Minimum variance beamforming

The MV beamformer can significantly improve the resolution of ultrasound image by updating a set of apodization weights for each imaging point in the image. The weight vector is obtained by minimizing the power of the beamformer output under the constraint that the desired signal scattered from the imaging point is passed without distortion.

In practical applications, the covariance matrix is unavailable and usually estimated with the aperture data. To get a good estimate of covariance matrix, spatial smoothing and temporal smoothing techniques are suggested to be applied [2]. Then, the covariance matrix ${\mathbf {R}}_\mathrm{MV}(p)$ can be estimated as follows:

3 $$\begin{aligned} {\mathbf {R}}_\mathrm{MV}(p) = \frac{\sum _{k=-K}^{K}{\sum _{l=1}^{N-L+1}{{\mathbf {x}}_{l}(p+k)}{{\mathbf {x}}^{H}_{l}(p+k)} }}{(2K+1)(N-L+1)}, \end{aligned}$$

where *L* is the subarray length, and $2K+1$ is the number of axial imaging points used for temporal smoothing. ${(\cdot )}^{H}$ stands for the conjugate transpose, and ${\mathbf {x}}_{l}=[{\mathbf {x}}_{l}, {\mathbf {x}}_{l+1}, ...{\mathbf {x}}_{l+L-1}]^{T}$ represents the *l*th subarray with a length of *L*. Note that, the index of imaging point *p* has been removed for simplicity in notation, such that ${\mathbf {R}}_\mathrm{MV} = {\mathbf {R}}_\mathrm{MV}(p)$ and ${\mathbf {x}}_{l} = {\mathbf {x}}_{l}(p)$.

To ensure the estimation robustness, the diagonal loading (DL) is applied on ${\mathbf {R}}_\mathrm{MV}$, in which a constant is added into the diagonal elements of the covariance before estimating [1]. The diagonal-loaded covariance matrix is calculated as

4 $$\begin{aligned} \hat{{\mathbf {R}}}_\mathrm{MV}={\mathbf {R}}_\mathrm{MV}+\varepsilon {\mathbf {I}}, \end{aligned}$$

where ${\mathbf {I}}$ is an $L \times L$ identity matrix and $\varepsilon$ is the diagonal loading factor. The $\varepsilon$ is set to $\triangle$ times the power ratio of added spatial noise to received signal, that is $\varepsilon =\triangle \times \mathrm {trace}\{{\mathbf {R}}\}$, where $\triangle$ is typically less than 1/*L* to ensure a well-conditioned covariance matrix [2].

The optimum weight vector of MV is obtained as

5 $$\begin{aligned} {\mathbf {w}}_\mathrm{MV}=\frac{\hat{{\mathbf {R}}}_\mathrm{MV}^{-1}\mathbf {a}}{\mathbf {a}^{H}\hat{{\mathbf {R}}}_\mathrm{MV}^{-1}\mathbf {a}}, \end{aligned}$$

where **a** represents the directional vector, and $(\cdot )^H$ donates the conjugate transpose. Since the data have been focused and delayed, **a** is a column vector of ones [1].

The beamformed output of MV is finally given as

6 $$\begin{aligned} \begin{aligned} Y_\mathrm {MV} = {\frac{1}{N-L+1} \sum _{l=1}^{N-L+1} { {{\mathbf {w}}}^H_\mathrm{MV} {\mathbf {x}}_{l}} }. \end{aligned} \end{aligned}$$

### Eigenspace-based MV

For eigenspace-based MV (ESBMV) beamformer, the covariance matrix $\hat{{\mathbf {R}}}_{\mathrm {MV}}$ from MV solution is first decomposed to the signal subspace and noise subspace as

7 $$\begin{aligned} \hat{{\mathbf {R}}}_{\mathrm {MV}}=\sum _{j=1}^{L}{\lambda _i}{{\mathbf {e}}_j}{{\mathbf {e}}^{H}_j}={{\mathbf {E}}_s}{\mathbf {\Lambda }_s}{{\mathbf {E}}^H_s}+{{\mathbf {E}}_n}{\mathbf {\Lambda }_n}{{\mathbf {E}}^H_n}, \end{aligned}$$

where ${\mathbf {\Lambda }_{s}}=diag(\lambda _{1}, ... ,\lambda _{j})$, ${\mathbf {\Lambda }_{n}}=diag(\lambda _{j+1}, ... ,\lambda _{L})$, and $\lambda _j$ is the eigen value of the matrix $\hat{{\mathbf {R}}}_{\mathrm {MV}}$. $\lambda _1 \ge \lambda _2 \ge \ldots \ge \lambda _{L}$ are the eigenvalues in descending order. The signal subspace ${\mathbf {E}}_s=[{\mathbf {e}}_1,...,{\mathbf {e}}_j]$, and the noise subspace ${\mathbf {E}}_n=[{\mathbf {e}}_{j+1},...,{\mathbf {e}}_{L}]$.

The eigen vector corresponding to $\lambda _j$ is used to construct the signal subspace $\mathbf{E} _s$, if ${\lambda _j \ge \gamma \lambda _{max}}$, where $\gamma$ is the threshold of eigenvalues and $\lambda _{max}$ is the largest eigenvalue. The weight vector of ESBMV is then obtained by projecting the MV weight vector into the signal subspace

8 $$\begin{aligned} {\mathbf {w}}_\mathrm{ESBMV} = {\mathbf {E}}_s{\mathbf {E}}^H_s{\mathbf {w}}_\mathrm{MV}. \end{aligned}$$

The beamformed output of ESBMV can be obtained according to (6) and (8).

### Generalized coherence factor

The generalized coherence factor (GCF) [18] is defined as the ratio of the spectral energy in a pre-specified low-frequency region (LFR) to the total energy. The GCF over the aperture at a given range is expressed as

9 $$\begin{aligned} W_{\mathrm {GCF}} = \frac{ \sum _{k \in [-M_0, M_0]}^{}{| s(k)|^2 } }{\sum _{k=-\frac{N}{2}}^{\frac{N}{2}-1}| {s(k)} | ^2}, \end{aligned}$$

where *s*(*k*) is the Fourier transform of ${\mathbf {x}}$, $M_0$ is the cut-off frequency, and $k \in [-\frac{N}{2}, -\frac{N}{2}+1 , ... ,\frac{N}{2}-1]$ is the spatial frequency index.

### Proposed method

We first form the CMSB to an adaptive weighting approach for MV, and then introduce adaptive diagonal reducing to the formulated weighting approach to further improve the imaging performance.

#### Covariance matrix-based statistical factor for MV

In CMSB, the reciprocals of the amplitude standard deviations (ASD) of the aperture data from all imaging points are normalized and square rooted to dynamically determine the subarray length for covariance estimation. The ASD of the aperture data is given as

10 $$\begin{aligned} \sigma = \sqrt{\frac{1}{N}\sum _{n=1}^{N}{({\mathbf {x}}_{n} - \bar{{\mathbf {x}}})^2} }, \end{aligned}$$

where $\bar{{\mathbf {x}}}=\frac{1}{N}\sum _{n=1}^{N}{{\mathbf {x}}_{n}}$. The normalized reciprocals of the ASD from all imaging points are

11 $$\begin{aligned} \varvec{\sigma '} = {\amalg {( \varvec{\sigma }^{-\frac{1}{3}}} ) }, \end{aligned}$$

where $$\amalg {(\cdot )}$$ is the normalization operation. The dynamic subarray length $$ L^{'}$$ for imaging point *p* is selected as

12 $$\begin{aligned} L^{'} = \lfloor \sigma ' \cdot L_{max} \rfloor , \end{aligned}$$

where $\lfloor \cdot \rfloor$ represents rounding down to the nearest integer, and $L_{max}$ represents the maximum subarray length. $L^{'}$ is set to 2 when $L^{'} \le 2$ to guarantee a matrix form.

The covariance matrix ${\mathbf {R}}$ estimated using the dynamic subarray length $L^{'}$ is then given as

13 $$\begin{aligned} \begin{aligned} {\mathbf {R}} = \frac{1}{N-L^{'}+1} \sum _{l=1}^{N-L^{'}+1}{\hat{{\mathbf {x}}}_{l}}{\hat{{\mathbf {x}}}^{H}_{l}} , \end{aligned} \end{aligned}$$

where $$\hat{{\mathbf {x}}}_{{l}}=[{\mathbf {x}}_{l}, {\mathbf {x}}_{l+1}, ... , {\mathbf {x}}_{l+L^{'}-1}]^T$$.

The estimated ${\mathbf {R}}$ is first rotary averaged and then diagonal reduced to reconstruct a modified covariance matrix. The rotary averaging on ${\mathbf {R}}$ is given as

14 $$\begin{aligned} \hat{\mathbf {\varvec{R}}} = \frac{1}{4} ({\mathbf {\varvec{R}}} + \mathbf {\varvec{J}}\mathbf {{\varvec{R}}}^T + \mathbf {\varvec{J}}\mathbf {{\varvec{R}}}\mathbf {\varvec{J}} + \mathbf {{\varvec{R}}}^T\mathbf {\varvec{J}}). \end{aligned}$$

The diagonal reducing is then performed on the rotary-averaged covariance matrix as

15 $$\begin{aligned} \widehat{\mathbf {\varvec{R}}} = \hat{\mathbf {\varvec{R}}} - \delta \hat{\mathbf {\varvec{R}}} \cdot \mathbf {\varvec{I}}, \end{aligned}$$

where $\delta$ is the diagonal reducing factor.

We here introduce an adaptive weighting approach for MV beamformer to improve the imaging performance. The pixel-based adaptive weighting named as covariance matrix-based statistical factor (CMSF) is defined as the mean-to-standard-deviation ratio (MSR) of the modified covariance matrix,

16 $$\begin{aligned} \begin{aligned} W_\mathrm {CMSF} = \frac{ \mathrm {E}[ \widehat{\mathbf {\varvec{R}}} ] }{ \mathrm {Std}[ \widehat{\mathbf {\varvec{R}}} ] },\\ \end{aligned} \end{aligned}$$

where $\mathrm {E}[ \widehat{\mathbf {\varvec{R}}} ]$ and $\mathrm {Std}[ \widehat{\mathbf {\varvec{R}}} ]$ are the mean and standard deviation of the coavariance matrix $\widehat{\mathbf {\varvec{R}}}$, respectively, wihich are calculated as

17 $$\begin{aligned} \begin{aligned} \mathrm {E}[ \widehat{\mathbf {\varvec{R}}} ] = \frac{1}{{L^{'}}^2} \sum _{k=1}^{L^{'}}\sum _{n=1}^{L^{'}} {\widehat{\mathbf {\varvec{R}}}_{k,n}},\\ \mathrm {Std}[ \widehat{\mathbf {\varvec{R}}} ] = {\sqrt{\frac{1}{{L^{'}}^2}\sum _{k=1}^{L^{'}}\sum _{n=1}^{L^{'}} (\widehat{\mathbf {\varvec{R}}}_{k,n} - \mathrm {E}[ \widehat{\mathbf {\varvec{R}}} ])^2} }. \end{aligned} \end{aligned}$$

The output of the proposed CMSF-weighted MV (CMSF-MV) is obtained according to (6) and (16) as

18 $$\begin{aligned} \begin{aligned} Y_\mathrm {CMSF-MV} = W_\mathrm {CMSF} \times Y_\mathrm {MV}. \end{aligned} \end{aligned}$$

**Fig. 8:**
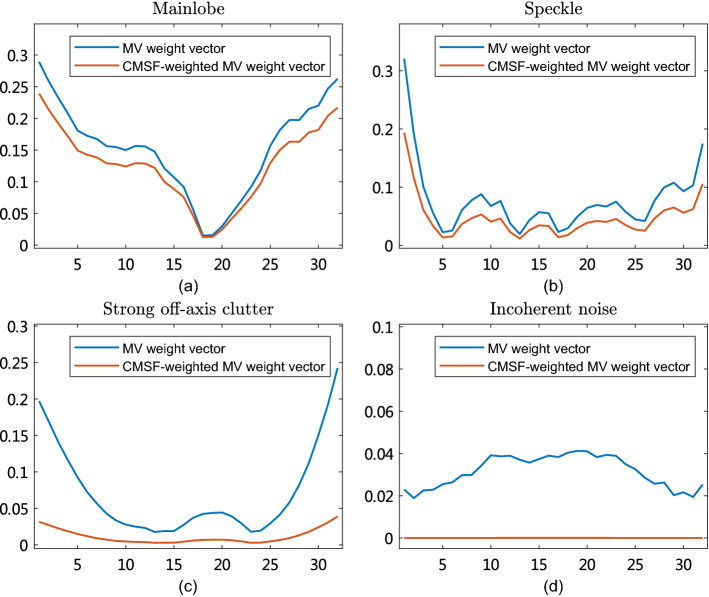
The weight vector with and without CMSF weighting in MV beamformer for **a** mainlobe, **b** speckle signals, **c** strong off-axis clutter, and **d** incoherent noise

In CMSF, the subarray lengths for strong off-axis clutter and incoherent noise are usually small and large, respectively; whereas, the subarray length for speckle signals is slightly smaller than that for coherent signals. Therefore, the estimated CMSF value for strong clutter is much lower than that of speckle signals and coherent signals, and this contributes to clutter suppression. Moreover, the CMSF value for incoherent noise is always very low, which contributes to reduce incoherent noise. Fig. 8 shows the weight vector with and without the proposed CMSF weighting in MV beamformer for mainlobe, speckle signals, strong off-axis clutter, and incoherent noise. It is seen that the CMSF-weighted MV weight vector for mainlobe and speckle retains to some extent compared with the MV weight vector, while that for strong off-axis clutter and incoherent noise is degraded dramatically. Thus, CMSF has the potential to reduce strong off-axis clutter and incoherent noise, thus resulting in improved resolution and contrast.

#### Covariance matrix-based statistical adaptive weighting for MV

For CMSF weighting, the resolution and speckle preservation will enhance as the diagonal reducing factor $\delta$ increases, whereas the contrast degrades. We hypothesize that selecting the pixel-based $\delta$ adaptively has the potential to overcome the tradeoff between contrast and speckle preservation. To this end, we introduce an adaptive diagonal reducing approach based on the aperture coherence to improve the imaging performance.

The CF weight is calculated by estimating the coherence of echo signals as

19 $$\begin{aligned} \begin{aligned} W_{\mathrm {CF}} = \frac{|\sum _{n=1}^{N} {{\mathbf {x}}_n}|^2}{N\sum _{n=1}^{N} {|{\mathbf {x}}_n|^2}} . \end{aligned} \end{aligned}$$

The value of CF equals 1 when signals are perfectly coherent, whereas it falls to 0 in the case of incoherent noise when signals are completely misaligned with each other. CF can effectively suppress off-axis signals but it tends to oversuppress incoherent speckle signals. Nevertheless, the intensity and smoothness of speckle texture generated by CF can be improved by using the normalized reciprocal of ASD (i.e., $\varvec{\sigma '}$) to adjust the value of CF.

We propose to adaptively select the diagonal reducing factor using $W_{\mathrm {CF}}$ and $\sigma '$ for each imaging point as

20 $$\begin{aligned} \begin{aligned} \delta _{ac} = W_{\mathrm {CF}}^{\sigma '} \times \delta _{max}, \end{aligned} \end{aligned}$$

where $\delta _{max}$ is the maximum diagonal reducing factor, and $\sigma '$ is the normalized reciprocal of the amplitude standard deviation from (11). Then the value of $\delta _{ac}$ is in the range of [0, $\delta _{max}$] according to (20).

The adaptively diagonal reduced covariance matrix using the pixel-based $\delta _{ac}$ is then given as

21 $$\begin{aligned} \widetilde{\mathbf {\varvec{R}}} = \hat{\mathbf {\varvec{R}}} - \delta _{ac} \hat{\mathbf {\varvec{R}}} \cdot {\mathbf {I}}. \end{aligned}$$

The pixel-based weight value of the proposed CMSAW is obtained by averaging the MSR of the $\widetilde{\mathbf {\varvec{R}}}$ as

22 $$\begin{aligned} \begin{aligned} W_\mathrm {CMSAW} = \frac{ \mathrm {E}[ \widetilde{\mathbf {\varvec{R}}} ] }{ \mathrm {Std}[ \widetilde{\mathbf {\varvec{R}}} ] }, \end{aligned} \end{aligned}$$

where $\mathrm {E}[ \widetilde{\mathbf {\varvec{R}}} ]$ and $\mathrm {Std}[ \widetilde{\mathbf {\varvec{R}}} ]$ are calculated according to (17) using $\widetilde{\mathbf {\varvec{R}}}$.

**Fig. 9:**
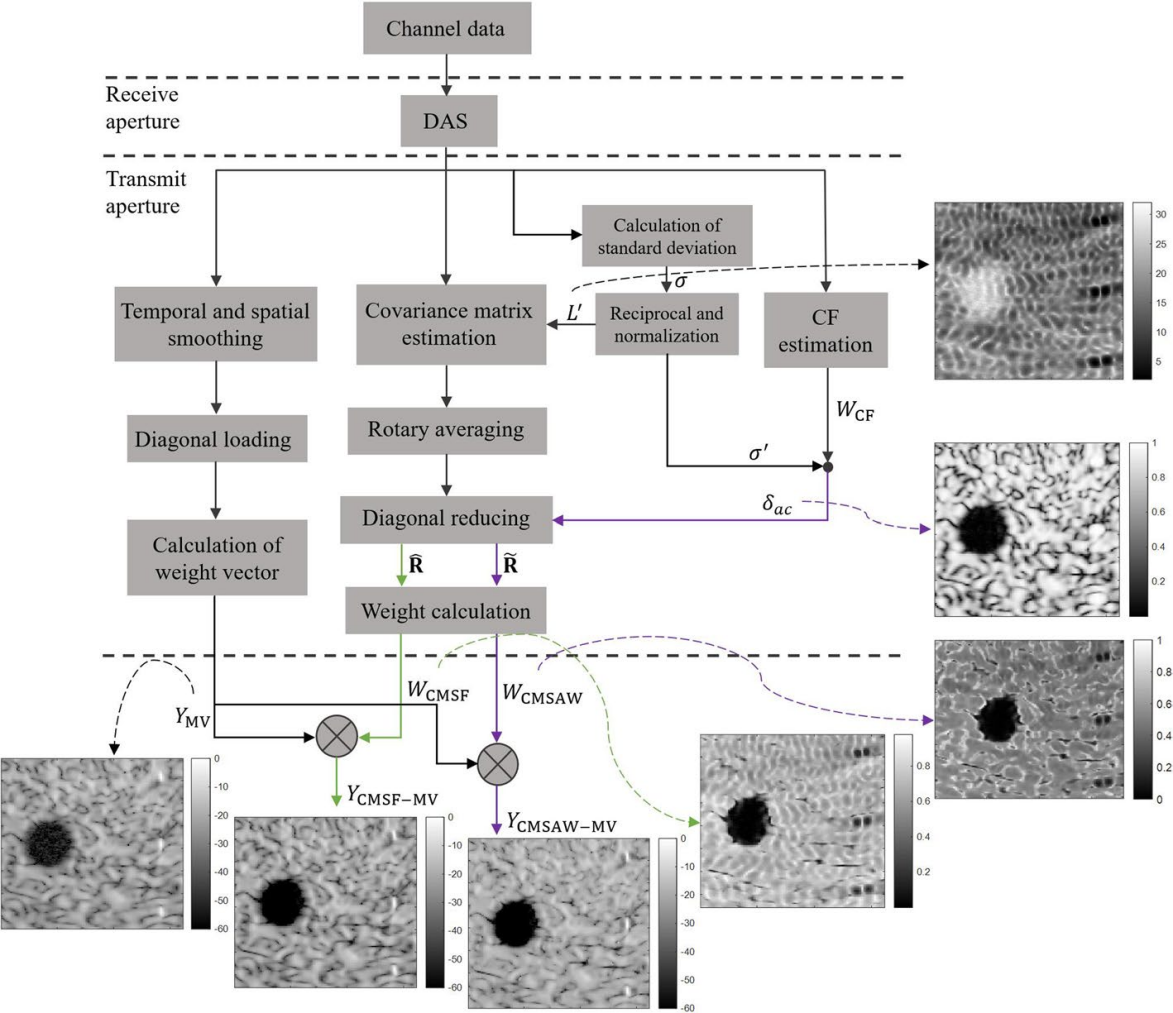
Workflow for the proposed CMSF-MV and CMSAW-MV

For mainlobe and incoherent noise, the estimated CF values are large and low, respectively, whereas the estimated normalized reciprocal of ASDs are low and large, respectively. As a result, the selected diagonal reducing factor $\delta _{ac}$ for mainlobe and incoherent noise are large and small. The estimated MSR value for incoherent noise is very small with a large subarray length and a low $\delta$, which leads to reduction of incoherent noise. Besides, the selected $\delta$ for speckle using Equation (23) is larger and lower than that for incoherent noise and mainlobe, respectively. According to the MSR for various targets as a function of $\delta$ in [30], the estimated MSR for speckle is close to or much larger than that for mainlobe, which can be seen in Fig. 9. This will lead to further preservation of speckle signals. In addition, the reduction of strong off-axis clutter remains because the smallest subarray length and a large $\delta$ generates a much lower MSR value compared to that for mainlobe. Therefore, the adaptive diagonal reducing based on aperture coherence has the potential for speckle preservation and noise reduction.

The output of CMSAW-weighted MV (CMSAW-MV) is finally obtained according to (6) and (22) as

23 $$\begin{aligned} \begin{aligned} Y_\mathrm {CMSAW-MV} = W_\mathrm {CMSAW} \times Y_\mathrm {MV}. \end{aligned} \end{aligned}$$

The diagram of the proposed methods to form an image is displayed in Fig. 9, and the brief implementation summary of the procedures for the proposed methods is as follows:

1. Calculate and compensate time delays to channel signals for each imaging point;
2. Synthesize the delayed channel data in receive aperture by directly summing, and get the receive-synthesized data;
3. Normalize the reciprocals of amplitude standard deviations from all imaging points using (11);
4. Estimate the covariance matrix with adaptive subarray length using (12)-(13), and then perform rotary averaging using (14);
5. Calculate the dynamic diagonal reducing factor $\delta _{ac}$ using (20);
6. Perform diagonal reducing with a constant $\delta$ using (15) and a dynamic $\delta _{ac}$ using (21), respectively, and get $\widehat{\mathbf {\varvec{R}}}$ and $\widetilde{\mathbf {\varvec{R}}}$;
7. Calculate the CMSF and CMSAW weight values using (16) and (22);
8. Use the CMSF value and CMSAW value to weight the MV beamformed output, respectively, using (18) and (23), and obtain the final CMSF-MV and CMSAW-MV beamformed outputs.

### Simulation and experimental setups

We evaluated the imaging performance of the proposed methods through simulation and experimental studies. The datasets were acquired using the synthetic aperture ultrasound imaging mode.

#### Simulation study

Simulation was performed using the Field II program [39, 40] to evaluate the imaging performance of the proposed methods. In the simulation study, a tissue-mimicking phantom with three point targets and a 5 mm diameter cyst target in a speckle-generating background was designed. The phantom with a volume size of $22\ \mathrm {mm} \times 0.5\ \mathrm {mm} \times 15 \ \mathrm {mm}$ was simulated by randomly distributing random amplitude scatters with a zero-mean Gaussian distribution, and the density of the phantom is 40 scatters per resolution cell. The SA dataset was acquired with a 15.44 mm linear array probe with 64 elements, 0.24 mm spacing with center frequency of 3.33 MHz. The sampling frequency was 40 MHz, and the speed of sound (SOS) was 1540 m/s. After the echo data were obtained, a Gaussian distributed noise with an SNR of 10 dB was added to the channel data prior to beamforming.

#### Phantom study

To validate the results from the simulation, we applied the proposed methods to experimental dataset. The complete dataset *geabr_0* was originally provided by Biomedical Ultrasound Laboratory (BUL) at the University of Michigan, available at https://www.k-space.org/temp/Ultrasound/. The SA dataset *geabr_0* were acquired using an experimental system with a 64-element, 0.24-mm pitch transducer array. The center frequency and sampling frequency were 3.33 MHz and 17.76 MHz, respectively, and the SOS was 1500 m/s. A finite impulse response bandpass filter was used to filter the channel data to reduce noise prior to beamforming.

Additionally, a tissue-mimicking phantom dataset *ats* was used to evaluate the imaging performance of the proposed methods on hypoechoic and hyperechoic cysts. The dataset *ats* was acquired from a region with different imaging targets in an ATS Model 539 tissue-mimicking phantom [36]. The complete dataset *rat_tumor* was provided by the Bioacoustics Research Lab (BRL) at the University of Illinois, available at www.brl.uiuc.edu/Projects/phase_aberration.php. The dataset was acquired with a 64-element and 0.315-mm pitch linear array excited at a 2.6-MHz center frequency, and the sampling rate was 25 MHz. The SOS was assumed to be 1450 m/s. The channel data were bandpass filtered in time and dip filtered in space–time prior to beamforming.

#### In vivo studies

In addition, we evaluated the imaging performance of the proposed methods on the rat mammary tumor study to show its effectiveness. The complete dataset *rat_tumor* was also provided by the BRL, available at www.brl.uiuc.edu/Projects/phase_aberration.php. The SA data were acquired from a rat mammary tumor with a 64-element and 0.315-mm pitch linear array excited at a 2.6 MHz center frequency. The sampling rate was 25 MHz, and the SOS was assumed to be 1500 m/s. The channel data were bandpass filtered in time and dip filtered in space–time prior to beamforming.

We also used a human heart dataset [5] to preliminary test and verify the feasibility of the proposed methods for heart imaging. The dataset was acquired using the Verasonics Vantage 256 system (Verasonics, Kirkland, WA, USA) with the Verasonics P4-2v 64-element, 0.3-mm pitch, phased array probe transmitting with a center frequency at 3 MHz and sampled at 11.904 MHz. With the help of the recovery technique [41], we recovered the complete SA dataset from the focused transmit beams. The heart data in the parasternal view contain 50 frames, and the data in frame 15 were used in this study.

#### Parameter settings in beamformers

In this study, the proposed CMSF-MV and CMSAW-MV were compared with DAS, MV, ESBMV, and GCF-weighted MV (GCF-MV). For DAS beamformer, the *f*-number was set to 0 in all studies and a rectangular window was applied to obtain a better resolution in simulation and experimental phantom study using the dataset *geabr_0*, and a hamming window was applied to obtain a better contrast in other studies. The subarray length *L* was set to *N*/2, $\triangle$ was set to 0.1/*L*, and the number of axial imaging points $2K+1$ was set to 9 in MV and ESBMV beamformers in all studies. The eigenvalue threshold $\gamma$ in ESBMV was set to 0.5 in simulation and experimental studies, and it was set to 0.3 in rat mammary tumor study and 0.1 in the human heart study. The cut-off frequency $M_0$ in GCF was set to 1 in all studies to achieve better speckle preservation as well as noise reduction. The $\delta$ in CMSF-MV and $\delta _{max}$ in CMSAW-MV were both set to 1 in simulation, experimental phantom study with dataset *geabr_0*, rat mammary tumor study, and human heart study, and were set to 0.5 in experimental phantom study with dataset *ats*.

#### Evaluation metrics

The resolution was measured by the lateral full-width at half-maximum (FWHM, $-6$ dB beam width) of a point target in the lateral distance. For cyst images, contrast ratio (CR) [20] was measured using

24 $$\begin{aligned} \mathrm {CR}=20\mathrm{log}_{10}\big(\frac{\mu _\mathrm {cyst}}{\mu _\mathrm {bck}}\big), \end{aligned}$$

where $\mu _\mathrm {cyst}$ and $\mu _\mathrm {bck}$ are the mean values (before log-compression) in the cyst and speckle region, respectively.

The generalized contrast-to-noise ratio (gCNR) [42], which is robust against dynamic range transformations, was assessed to evaluate the lesion detectability in this study. The gCNR is defined as

25 $$\begin{aligned} \mathrm {gCNR}=1-\int min{ \{ p_\mathrm {cyst}(x), p_\mathrm {bck}(x) \} }\, dx, \end{aligned}$$

where $p_\mathrm {cyst}(x)$ and $p_\mathrm {bck}(x)$ are the probability density functions (PDFs) of pixel intensity inside the cyst and speckle regions, respectively.

The speckle signal-to-noise ratio (sSNR) [20] was measured to evaluate the speckle quality. It is calculated as

26 $$\begin{aligned} \mathrm {sSNR}=\frac{\mu _\mathrm {bck}}{\sigma _\mathrm {bck}}, \end{aligned}$$

where $\sigma _\mathrm {bck}$ is the standard deviation in the speckle region.

**Table 1:** Average FWHM, CR, gCNR, and sSNR of simulated images formed using different methods

Methods	FWHM (mm)	CR (dB) gCNR sSNR
DAS	0.86	− 29.4 0.88 1.87
MV	0.31	− 32.0 0.84 1.77
ESBMV	0.30	− 44.6 0.85 1.82
GCF-MV	0.30	− 48.5 0.73 1.54
CMSF-MV	0.19	− 51.0 0.82 1.62
CMSAW-MV	0.20	− 51.0 0.93 2.03

**Table 2:** Lateral FWHM, CR, gCNR, and sSNR of three anechoic cyst targets and one hyperechoic target at depths of 48, 68, 88, and 48 mm, respectively, of experimental images formed from dataset *geabr_0* using different methods

Methods	FWHM (mm)	CR (dB) gCNR sSNR
DAS	2.38	− 19.4/− 21.7/− 17.0/11.5 0.42/0.75/0.50/0.80 1.95/2.09/2.05/1.84
MV	0.47	− 17.3/− 18.6/ - 14.7/9.42 0.19/0.49/0.34/0.40 1.70/1.80/1.99/1.77
ESBMV	0.49	− 28.5/− 32.2/− 23.5/8.61 0.17/0.48/0.35/0.37 1.73/1.79/1.92/1.80
GCF-MV	0.47	− 32.0/− 38.8/− 33.2/11.4 0.10/0.31/0.10/0.55 1.32/1.41/1.19/1.40
CMSF-MV	0.37	− 31.8/− 38.5/− 34.8/7.58 0.55/0.79/0.59/0.39 1.48/1.64/1.39/1.57
CMSAW-MV	0.37	$-33.9/-39.5/-35.9$/6.41 0.77/0.94/0.72/0.41 1.82/2.07/1.47/1.84

**Table 3:** CR, gCNR, and sSNR of anechoic, hypoechoic, and hyperechoic cysts at depths of 22, 114.5, and 39.2 mm, respectively, in phantom experimental images formed from dataset *ats* using different methods

Methods	CR (dB)	gCNR	sSNR
DAS	− 23.9/− 6.6/10.9	0.81/0.24/0.75	2.16/1.88/2.06
MV	− 22.0/− 4.6/9.4	0.93/0.37/0.66	2.06/1.95/2.01
ESBMV	− 31.6/− 3.5/8.9	0.93/0.24/0.59	1.99/1.51/1.89
GCF-MV	− 35.8/− 9.2/10.3	0.85/0.25/0.62	1.75/1.54/1.60
CMSF-MV	− 37.2/− 8.7/8.2	0.95/0.43/0.60	1.97/1.36/1.74
CMSAW-MV	**− 38.9/− 9.8**/8.0	**0.98/0.46**/0.63	**2.36**/1.36/1.96

**Table 4:** CR, gCNR, and sSNR of rat mammary tumor images formed using different methods

Methods	CR (dB)	gCNR	sSNR
DAS	− 25.6	0.80	0.69
MV	− 22.7	0.69	0.74
ESBMV	− 24.3	0.42	0.74
GCF-MV	− 39.5	0.47	0.70
CMSF-MV	− 38.5	0.74	0.89
CMSAW-MV	**− 40.8 **	**0.85**	**1.17**

**Table 5:** CR, gCNR, and sSNR of human heart images formed using different methods

Methods	CR (dB) gCNR sSNR
DAS	− 34.7 0.76 1.62
MV	− 31.5 0.84 1.83
ESBMV	− 29.6 0.65 1.36
GCF-MV	− 41.9 0.57 1.28
CMSF-MV	− 40.3 0.88 1.78
CMSAW-MV	**− 43.2** ** 0.96** **2.14**

## Data Availability

Data are available upon request.

## Abbreviations

SA: Synthetic aperture
DAS: Delay-and-sum
MV: Minimum variance
ESBMV: Eigenspace-based minimum variance
GCF: Generalized coherence factor
CMSB: Covariance matrix-based statistical beamforming
MSR: Mean-to-standard-deviation ratio
CMSF: Covariance matrix-based statistical factor
CMSAW: Covariance matrix-based statistical adaptive weighting
FWHM: Full-width at half-maximum
CR: Contrast ratio
gCNR: Generalized contrast-to-noise ratio
sSNR: Speckle signal-to-noise ratio
